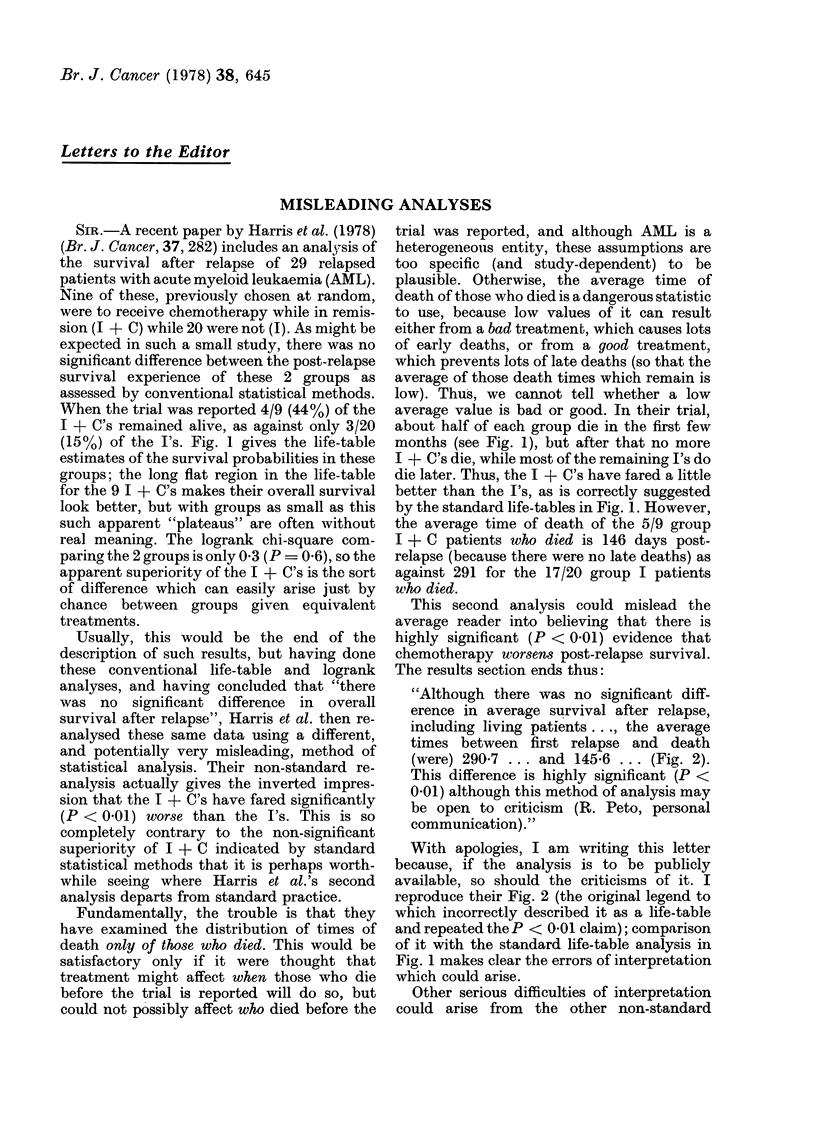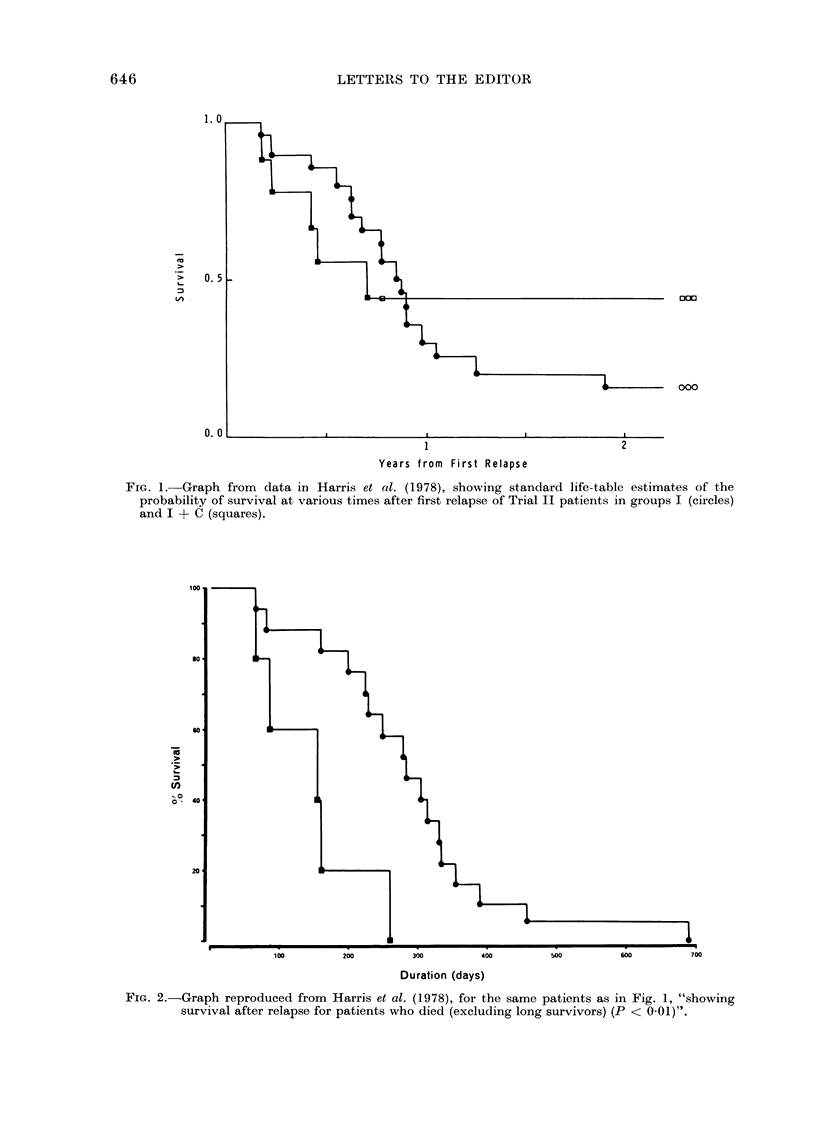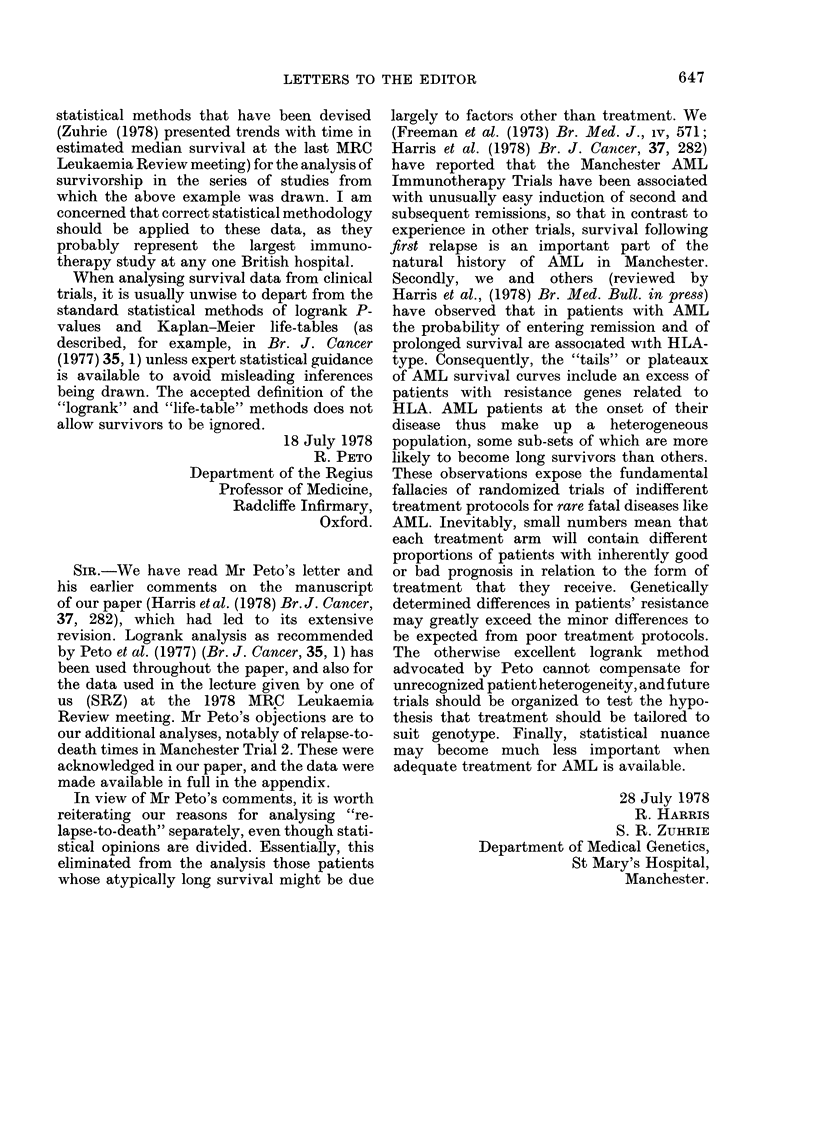# Misleading analyses.

**DOI:** 10.1038/bjc.1978.265

**Published:** 1978-11

**Authors:** R. Peto


*Br. J. Cancer* (1978) **38**, 645

### *Letters to the Editor*

### MISLEADING ANALYSES

SIR.—A recent paper by Harris *et al.* (1978) (*Br. J. Cancer*, **37**, 282) includes an analysis of the survival after relapse of 29 relapsed patients with acute myeloid leukaemia (AML). Nine of these, previously chosen at random, were to receive chemotherapy while in remission (I + C) while 20 were not (I). As might be expected in such a small study, there was no significant difference between the post-relapse survival experience of these 2 groups as assessed by conventional statistical methods. When the trial was reported 4/9 (44%) of the I + C's remained alive, as against only 3/20 (15%) of the I's. Fig. 1 gives the life-table estimates of the survival probabilities in these groups; the long flat region in the life-table for the 9 I + C's makes their overall survival look better, but with groups as small as this such apparent "plateaus" are often without real meaning. The logrank chi-square comparing the 2 groups is only 0·3 ($P = 0{\cdot}6$), so the apparent superiority of the I + C's is the sort of difference which can easily arise just by chance between groups given equivalent treatments.

Usually, this would be the end of the description of such results, but having done these conventional life-table and logrank analyses, and having concluded that "there was no significant difference in overall survival after relapse", Harris *et al.* then reanalysed these same data using a different, and potentially very misleading, method of statistical analysis. Their non-standard reanalysis actually gives the inverted impression that the I + C's have fared significantly ($P < 0{\cdot}01$) *worse* than the I's. This is so completely contrary to the non-significant superiority of I + C indicated by standard statistical methods that it is perhaps worthwhile seeing where Harris *et al.*'s second analysis departs from standard practice.

Fundamentally, the trouble is that they have examined the distribution of times of death *only of those who died.* This would be satisfactory only if it were thought that treatment might affect *when* those who die before the trial is reported will do so, but could not possibly affect *who* died before the trial was reported, and although AML is a heterogeneous entity, these assumptions are too specific (and study-dependent) to be plausible. Otherwise, the average time of death of those who died is a dangerous statistic to use, because low values of it can result either from a *bad* treatment, which causes lots of early deaths, or from a *good* treatment, which prevents lots of late deaths (so that the average of those death times which remain is low). Thus, we cannot tell whether a low average value is bad or good. In their trial, about half of each group die in the first few months (see Fig. 1), but after that no more I + C's die, while most of the remaining I's do die later. Thus, the I + C's have fared a little better than the I's, as is correctly suggested by the standard life-tables in Fig. 1. However, the average time of death of the 5/9 group I + C patients *who died* is 146 days post-relapse (because there were no late deaths) as against 291 for the 17/20 group I patients *who died.*

This second analysis could mislead the average reader into believing that there is highly significant ($P < 0{\cdot}01$) evidence that chemotherapy *worsens* post-relapse survival. The results section ends thus:

> "Although there was no significant difference in average survival after relapse, including living patients . . ., the average times between first relapse and death (were) 290·7 . . . and 145·6 . . . (Fig. 2). This difference is highly significant ($P < 0{\cdot}01$) although this method of analysis may be open to criticism (R. Peto, personal communication)."

With apologies, I am writing this letter because, if the analysis is to be publicly available, so should the criticisms of it. I reproduce their Fig. 2 (the original legend to which incorrectly described it as a life-table and repeated the $P < 0{\cdot}01$ claim); comparison of it with the standard life-table analysis in Fig. 1 makes clear the errors of interpretation which could arise.

Other serious difficulties of interpretation could arise from the other non-standard

FIG. 1.—Graph from data in Harris *et al.* (1978), showing standard life-table estimates of the probability of survival at various times after first relapse of Trial II patients in groups I (circles) and I + C (squares).

FIG. 2.—Graph reproduced from Harris *et al.* (1978), for the same patients as in Fig. 1, "showing survival after relapse for patients who died (excluding long survivors) ($P < 0{\cdot}01$)".

statistical methods that have been devised (Zuhrie (1978) presented trends with time in estimated median survival at the last MRC Leukaemia Review meeting) for the analysis of survivorship in the series of studies from which the above example was drawn. I am concerned that correct statistical methodology should be applied to these data, as they probably represent the largest immunotherapy study at any one British hospital.

When analysing survival data from clinical trials, it is usually unwise to depart from the standard statistical methods of logrank *P*-values and Kaplan–Meier life-tables (as described, for example, in *Br. J. Cancer* (1977) **35**, 1) unless expert statistical guidance is available to avoid misleading inferences being drawn. The accepted definition of the "logrank" and "life-table" methods does not allow survivors to be ignored.

18 July 1978

R. Peto
Department of the Regius
Professor of Medicine,
Radcliffe Infirmary,
Oxford.